# Infant, Neonatal, and Post-neonatal Mortality in Greece: A Nationwide Time-Trend Analysis

**DOI:** 10.7759/cureus.61418

**Published:** 2024-05-31

**Authors:** Nikolaos Vlachadis, Nikolaos Loukas, Nikolaos Antonakopoulos, Dionysios Vrachnis, Athanasios Zikopoulos, Sofoklis Stavros, Nikolaos Machairiotis, Maria Siori, Petros Drakakis, Nikolaos Vrachnis

**Affiliations:** 1 Department of Obstetrics and Gynecology, General Hospital of Messinia, Kalamata, GRC; 2 Third Department of Obstetrics and Gynecology, National and Kapodistrian University of Athens, Medical School, Attiko Hospital, Athens, GRC; 3 Department of Obstetrics and Gynecology, University of Patras, Rio Hospital, Patras, GRC; 4 Primary Health Center of Byron, National Health System of Greece, Athens, GRC

**Keywords:** economic crisis, trend analysis, greece, post-neonatal mortality, neonatal mortality, infant mortality rate (imr), infant mortality

## Abstract

Introduction: Infant mortality is a crucial perinatal measure and is also regarded as an important public health indicator. This study aimed to comprehensively present time trends in infant, neonatal, and post-neonatal mortality in Greece.

Methods: The annual infant mortality rate (IMR), the neonatal mortality rate (NMR), and the post-neonatal mortality rate (PNMR) were calculated based on official national data obtained from the Hellenic Statistical Authority, spanning 67 years from 1956 to 2022. The time trends of the mortality rates were evaluated using joinpoint regression analysis, and the annual percent changes (APC) and the overall average annual percent change (AAPC) were calculated with a 95% confidence interval (95% CI).

Results: The IMR exhibited accelerating declines over more than 50 years, with an APC of −1.9 (−2.8 to −1.0) from 1956 to 1968, −5.4 (−5.6 to −5.2) from 1968 to 1999, and −7.3 (−8.9 to −5.7) between 1999 and 2008. In 2008, IMR reached its all-time low of 2.7 per 1,000 live births, down 16.6-fold from its peak at 44.1 per 1,000 live births in 1957. This improving trend was reversed following the onset of the economic crisis in the country, leading to a 57% increase in IMR from 2008 to 2016, with an upward trend APC of 3.4 (1.2 to 5.5). In the recent period 2016-2022, there was an improvement with an APC of −3.7 (−6.2 to −1.1), resulting in an IMR of 3.1 per 1,000 live births in 2022. The decrease in IMR was estimated to have prevented 209,109 infant deaths in the country from 1958 to 2022. From 1956 to 2022, the IMR decreased with an AAPC of −3.9 (−4.3 to −3.4), while the PNMR saw a decline with an AAPC of −4.5 (−5.1 to −3.9) and the NMR with an AAPC of −3.2 (−3.7 to −2.6).

Conclusion: Greece achieved an impressive decrease in infant mortality rates, but this progress was halted and completely reversed during the economic crisis. Although there have been some recent improvements after the country's economic recovery, the rates have yet to reach pre-crisis levels.

## Introduction

Infant mortality refers to the probability of death during the first year of life and is expressed by the infant mortality rate (IMR), defined as the annual number of deaths in children aged less than one year per 1000 live births. The IMR is a central indicator for perinatal medicine but also a key measure for public health and overall population well-being because it correlates with socio-economic conditions and economic development, as well as the quality of health services in a country [1-7].

Disparities in infant mortality rates can indicate inequalities in access to healthcare, education, and other resources. Identifying and addressing these disparities can improve overall health outcomes for all members of the community. Moreover, the majority of infant deaths are preventable with access to quality prenatal care and public health measures, and monitoring the IMR can help identify gaps in these services and guide targeted interventions to reduce the burden of newborn deaths [8-10].

IMR is disaggregated into two categories: neonatal mortality rate (NMR), which includes deaths at <28 days of age, and post-neonatal mortality rate (PNMR), which refers to deaths of infants aged 28 to 364 days. NMR is a significant contributor to infant mortality rates since the neonatal period is the most vulnerable time for a child, and newborn deaths tend to cluster in the first few days of life [8,11].

Over the last few decades, there has been a substantial reduction in infant mortality worldwide, with the IMR dropping by more than half, from 65 deaths per 1,000 live births in 1990 to 29 deaths per 1,000 live births in 2018, and the annual infant deaths from 8.7 to 4.0 million, respectively. However, significant inequalities remain, and the probability of a child dying before their first birthday was most prevalent in Africa, with a rate of 52 deaths per 1,000 live births, which is more than seven times higher than the rate of 7 deaths per 1,000 live births in Europe [4].

The aim of the present study was to provide a comprehensive analysis of longitudinal trends in infant mortality in Greece at the national level by presenting the available data since 1956.

## Materials and methods

Publicly available official national data regarding infant, neonatal, and post-neonatal deaths, as well as live births in Greece, were retrieved from the Hellenic Statistical Authority, covering 67 years from 1956 to 2022.

For each year of the above period, we calculated the IMR as the number of deaths before the completion of one year of life, the NMR as the number of deaths before 28 days of life, and the PNMR as the number of deaths at 28 to 364 days of life, per 1,000 live births.

Trends in mortality rates were assessed using Joinpoint regression software (Surveillance Research Program, National Cancer Institute, Bethesda, Maryland, United States of America) to identify years at which significant changes in mortality trends occurred. The maximum number of allowed periods was set at 5, the annual percentage changes (APC) between joinpoint years were calculated with a 95% confidence interval (95% CI), and the level of statistical significance was set at p < 0.05. An APC was computed for each time segment between two joinpoints, and an overall average annual percent change (AAPC) was used as a summary measure of the trend, computed as a weighted average of the APCs over the whole examined period.

The number of averted infant deaths due to the decline in the IMR during 1958-2022 was estimated by the differences between the annual number of infant deaths that would have occurred if the IMR of the year 1957 (period high) had remained unchanged and the annual observed number of infant deaths.

## Results

**Figure 1:**
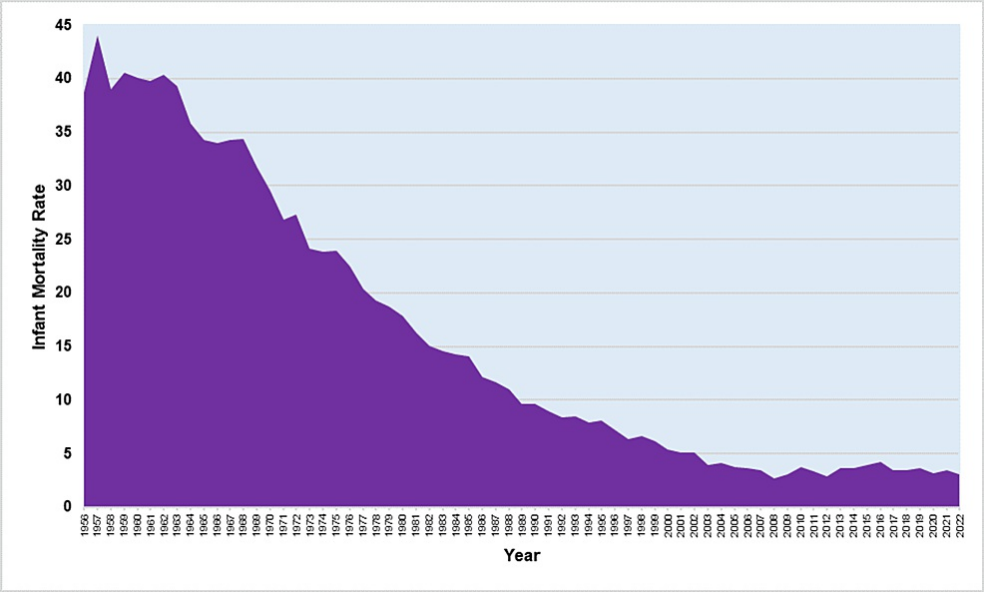
Infant mortality rate (per 1,000 live births) in Greece, 1956-2022.

Between 1956 and 2022, Greece recorded a total of 149,573 infant deaths and 8,144,472 live births, resulting in an overall IMR of 18.4 per 1,000 live births. The IMR decreased remarkably over these years, showing a 16.6-times reduction (94%) from a peak of 44.1 per 1,000 live births in 1957 to an all-time low of 2.7 per 1,000 live births in 2008 (Figure 1).

**Figure 2:**
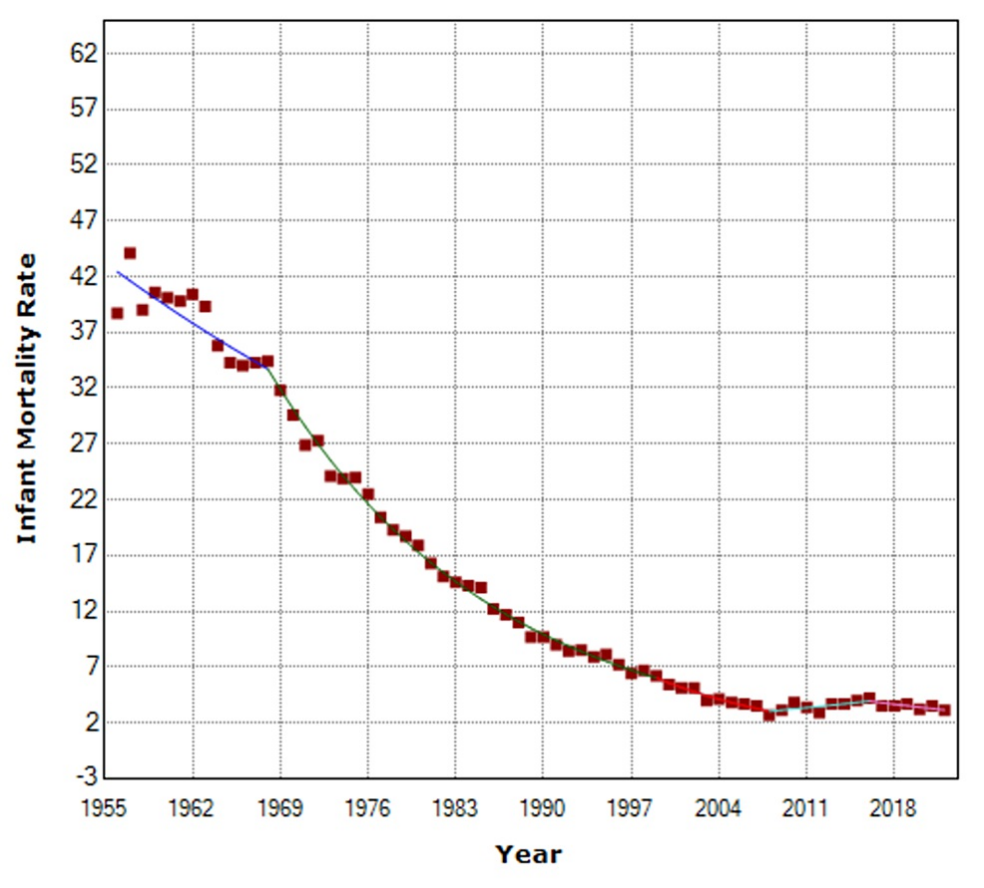
Time trends in infant mortality rates in Greece, 1956-2022.

**Figure 3:**
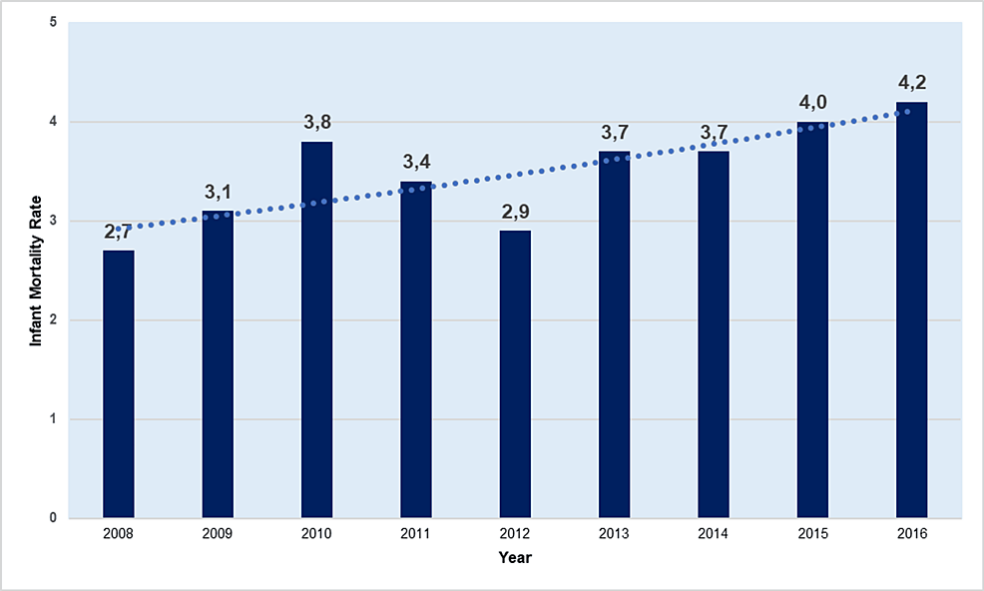
Infant mortality rate (per 1,000 live births) in Greece, 2008-2016.

The AAPC of the IMR reduction was −3.9 (95% CI: −4.3 to −3.4) for the whole period 1956-2022. There was an accelerating decline in IMR with APC = −1.9 (95% CI: −2.8 to −1.0) from 1956 to 1968, −5.4 (95% CI: −5.6 to −5.2) from 1968 to 1999, and −7.3 (95% CI: −8.9 to −5.7) from 1999 to 2008. However, from 2008 to 2016, there was an IMR increase attributed to the economic crisis, with an APC of 3.4 (95% CI: 1.2 to 5.5) and an overall increase of 57%, from 2.7 per 1,000 live births in 2008 to 4.2 per 1,000 live births in 2016. In the recent period 2016-2022, the improvement in IMR continued with APC = −3.7 (95% CI: −6.2 to −1.1) and a 25% decrease to 3.1 per 1,000 live births in 2022, 12.9 times down from the historic high of 1957 (Figures 2-3, Table 1).

**Table 1:** Time trends in infant mortality rates in Greece, 1956-2022. APC: annual percentage change.

Segment	APC	95% Confidence Interval	P-value
1956–1968	−1.9	−2.8 to −1.0	<0.001
1968–1999	−5.4	−5.6 to −5.2	<0.001
1999–2008	−7.3	−8.9 to −5.7	<0.001
2008–2016	3.4	1.2 to 5.5	0.002
2016–2022	−3.7	−6.2 to −1.1	0.006

**Figure 4:**
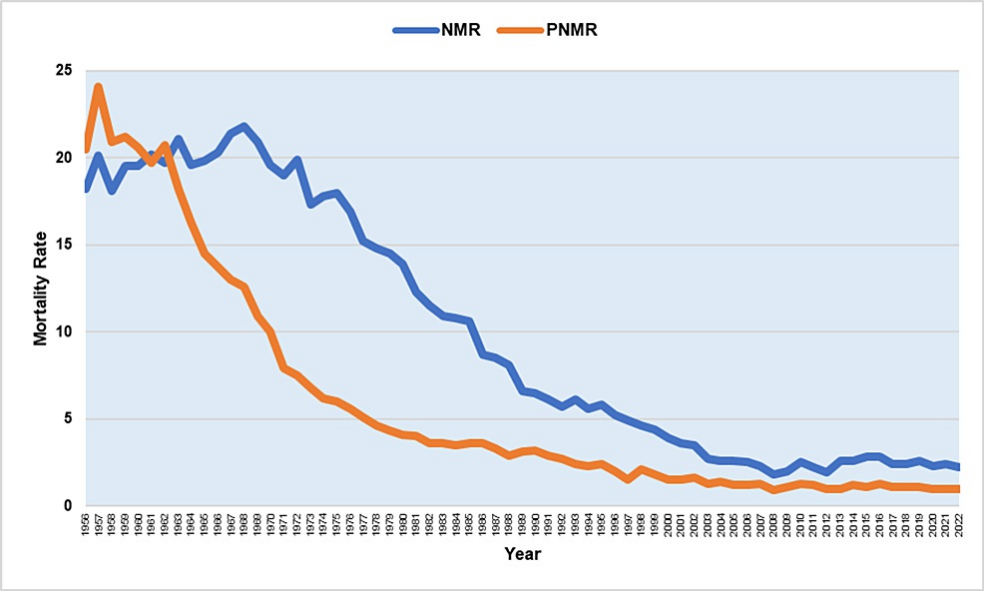
Neonatal mortality rate (NMR) and post-neonatal mortality rate (PNMR) in Greece (per 1,000 live births), 1956-2022.

Figure 4 shows the NMR and PNMR for the period 1956-2022.

The PNMR maximum (24.1 per 1,000 live births) was in 1957, while the NMR peak was recorded in 1968 (21.8 per 1,000 live births). Historical minimums for both rates were in 2008, with NMR = 1.8 per 1,000 live births, down 92%, or 12.2 times, compared with 1968, and PNMR = 0.9 per 1,000 live births, down 96%, or 28 times, from 1957. Initially, post-neonatal mortality was higher than neonatal mortality; however, NMR has been consistently superior to PNMR since 1962.

**Figure 5:**
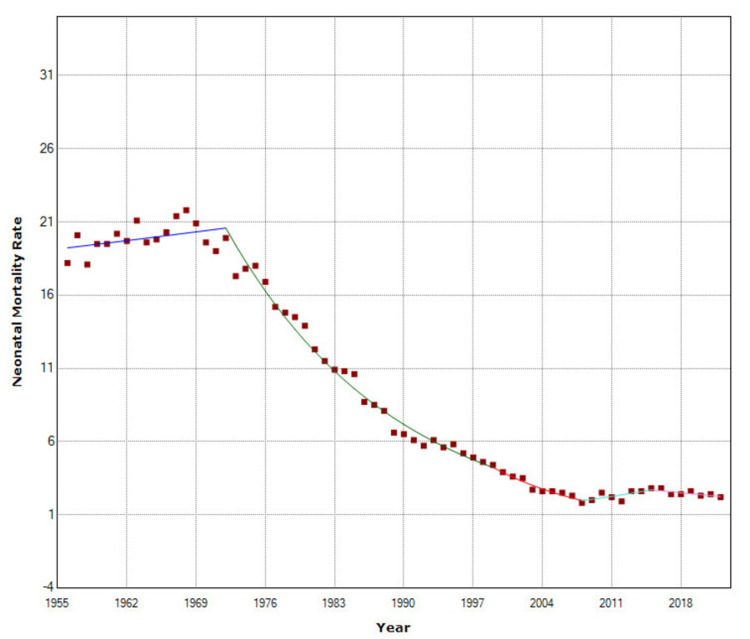
Time trends in neonatal mortality rate in Greece, 1956-2022.

**Figure 6:**
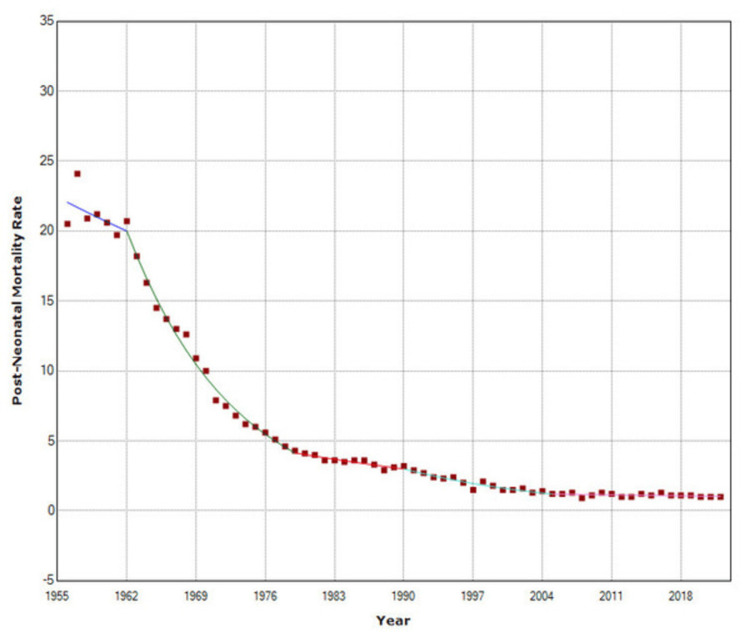
Time trends in post-neonatal mortality in Greece, 1956-2022.

NMR remained unchanged in the period 1956-1972 (p = 0.259) and then decreased at APC = −5.7 (95% CI: −6.0 to −5.4) during 1972-1999 and faster in the period 1999-2008 (APC = −8.2, 95% CI: −10.1 to −6.2). This was followed by an upward trend (APC = 4.7, 95% CI: 1.3 to 8.2) during the financial crisis, with a 57% increase by 2016, from 1.8 to 2.8 per 1,000 live births, and finally, a marginally statistically significant improvement from 2016 to 2022 (APC = −2.6, 95% CI: −5.1 to −0.0) (p = 0.049). PNMR was stable until 1962 (p = 0.418), while the most rapidly declining period was from 1962 to 1979 (APC = −8.8, 95% CI: −9.7 to −8.0). The improvement continued at a slower rate in the period 1979-1990 (APC = −2.9, 95% CI: −4.7 to −1.1) and accelerated again from 1990 to 2005 (APC = −5.9, 95% CI: −7.0 to −4.9). Since 2005, a marginally statistically significant improvement trend has emerged (APC = −0.8, 95% CI: −1.7 to −0.0) (p = 0.046). From 1956 to 2022, PNMR exhibited steeper AAPC than NMR [−4.5 (95% CI: −5.1 to −3.9) versus −3.2 (95% CI: −3.7 to −2.6), respectively] (Figures 5-6 and Tables 2-3).

**Table 2:** Time trends in neonatal mortality rates in Greece, 1956-2022. APC: annual percentage change.

Segment	APC	95% Confidence Interval	P-value
1956–1972	0.4	−0.3 to 1.2	0.259
1972–1999	−5.7	−6.0 to -5.4	<0.001
1999–2008	−8.2	−10.1 to −6.2	<0.001
2008–2015	4.7	1.3 to 8.2	0.007
2015–2022	−2.6	−5.1 to −0.0	0.049

**Table 3:** Time trends in post-neonatal mortality in Greece, 1956-2022. APC: annual percentage change.

Segment	APC	95% Confidence Interval	P-value
1956–1962	−1.6	−5.5 to 2.4	0.418
1962–1979	−8.8	−9.7 to -8.0	<0.001
1979–1990	−2.9	−4.7 to -1.1	0.002
1990–2005	−5.9	−7.0 to -4.9	<0.001
2005–2022	−0.8	−1.7 to -0.0	0.046

**Figure 7:**
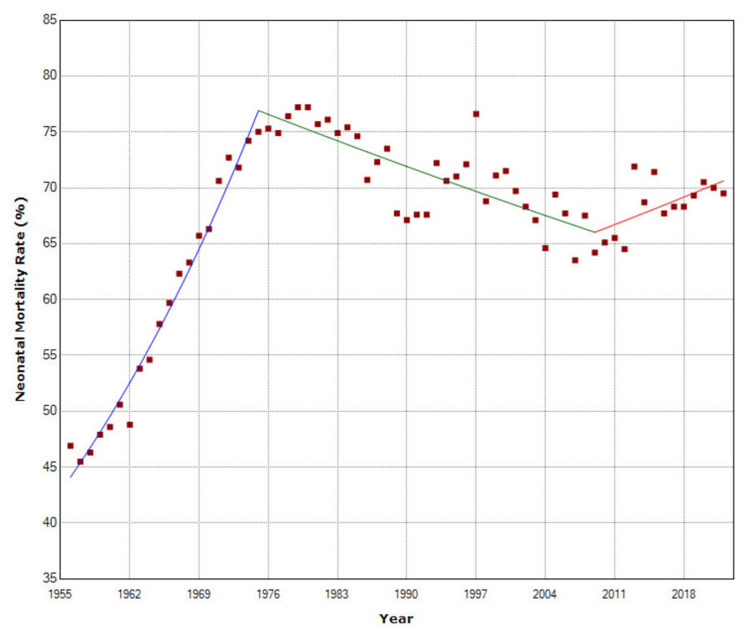
Trends in neonatal mortality (%) of infant mortality in Greece, 1956-2022.

In the total period 1956-2022, of the infant deaths, 92,596 were neonatal deaths (61.9%), and 56,977 were post-neonatal deaths (38.1%). Neonatal mortality as a proportion of infant mortality ranged from a minimum of 46.9% in 1956 to an all-time high of 77.2% in 1980 (64% increase), with APC = 3.0 (95% CI: 2.7 to 3.2) from 1956 to 1975, a downward trend for the period 1975-2009 (APC = −0.4, 95% CI: −0.6 to −0.3), and again a rising trend from 2009 to 2022 (APC = 0.5, 95% CI: 0.1 to 1.0), with overall AAPC = 0.7 (95% CI: 0.6 to 0.9). In 2022, the share of neonatal deaths in total infant deaths was 69.5% (Figure 7, Table 4).

**Table 4:** Time trends in neonatal mortality (%) of infant mortality in Greece, 1956-2022. APC: annual percentage change.

Segment	APC	95% Confidence Interval	P-value
1956–1975	3.0	2.7 to 3.2	<0.001
1975–2009	−0.4	−0.6 to −0.3	<0.001
2009–2022	0.5	0.1 to 1.0	0.034

**Figure 8:**
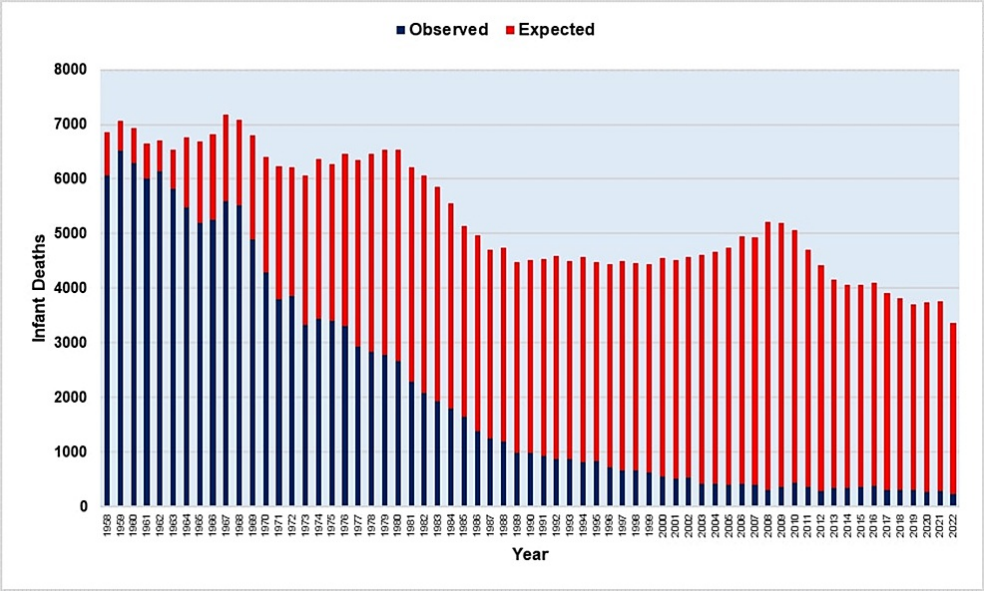
Estimated number of infant deaths averted (red-colored area) due to the improvement in infant mortality rates in Greece, 1958-2022.

Total annual infant deaths decreased by 28.8-fold, or 97%, from 6,884 in 1957 to a historical minimum of 239 in 2022. An estimated 53% of the decrease in infant deaths is attributable to the decline in live births, whereas the reduction in infant mortality drove 47%. The IMR decrease resulted in 209,109 estimated infant deaths from 1958 to 2022 (Figure 8).

**Figure 9:**
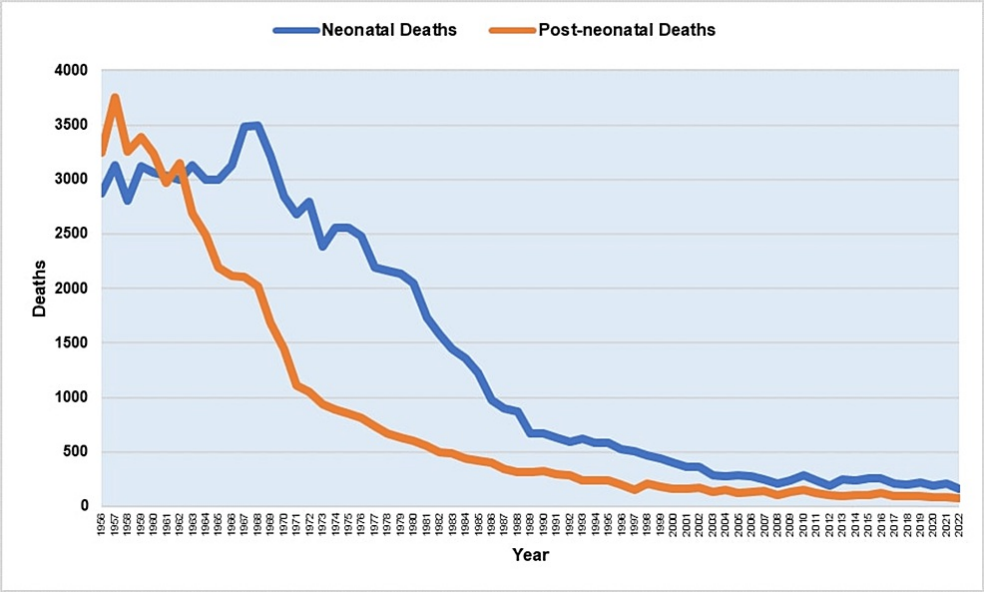
Neonatal and post-neonatal deaths in Greece, 1956-2022.

The maximum number of neonatal deaths was 3,494 in 1968, which declined 21-fold to an all-time low of 166 deaths in 2022, whereas post-neonatal deaths peaked at 3,752 in 1957, which was shrunk by a factor of 51.4 to a historical low of 73 deaths in 2022 (Figure 9).

## Discussion

The present study used official national data to highlight the longitudinal trends in infant and neonatal mortality in the Greek population over almost seven decades. Our findings show evidence that a remarkable reduction in infant mortality was achieved in Greece; however, the improvement in rates was interrupted and fully reversed during the economic recession, and despite the recent downward trend, the country is still struggling to restore pre-crisis levels.

The best-fitting model of the joinpoint analysis highlighted five sub-periods for IMR trends. For more than 50 years, IMR declined at an increasing annual rate, by 1.9% until 1968 and by 5.4% between 1968 and 1999, while the pace quickened to 7.3% per year during 1999-2008. These long favorable trends were dramatically reversed due to the economic crisis, with a statistically significant upward trend from 2008 to 2016 at an annual pace of 3.4%. An APC of 3.7 has restored the decline in IMR since 2016. In 2022, IMR was 12.9-fold lower as compared with the peak values of 1957, while infant deaths were 28.8-fold reduced, reflecting the dramatic parallel plummeting of birth rates [12].

An improvement in NMR was observed after 1972. The decrease occurred at an average annual rate of 5.7% between 1972 and 1999 and quickened to a steep 8.2% during the pre-crisis period of 1999-2008. In the financial crisis years 2008-2016, NMR had a rising trend of 4.7% per year, while a resumption of the downward trend observed since 2016 has been marginally statistically significant.

A rapid decreasing trend was seen for PNMR between 1962 and 1979 (APC = −8.8), which slowed down from 1979 to 1990 (APC = −2.9) and was accelerated again during 1990-2005 with an average annual decline of 5.9%, whereas since 2005 a slow marginally statistically significant downward trend has emerged.

From 1956 to 2022, the decline in PNMR was greater than that in NMR, with an average annual rate of 4.5% versus 3.2%, respectively. The improvement of NMR occurred with a lag period of approximately a decade, so the decreasing trend of PNMR became statistically significant after 1962 and that of NMR after 1972. PNMR saw a faster decline in the 1960s and 1970s, and the share of neonatal deaths among infant deaths peaked in 1980, while the improvement of NMR was superior to NMR from then on, despite a slight rising trend for the neonatal deaths proportion after 2009. The different patterns of longitudinal trends probably reflect epidemiological differences in etiology since neonatal deaths are mainly linked to gaps in the provision of perinatal care, while post-neonatal deaths are mostly associated with deteriorating socioeconomic conditions [13].

Time-trend analysis showed that 2008 was a landmark year in the overall evolution of infant mortality in Greece. In this year, the country saw historically low values in infant, neonatal, and post-neonatal mortality rates at 2.7, 1.8, and 0.9 deaths per 1,000 live births, respectively. However, the onset of the economic crisis turned IMR on an upward trend, essentially driven by an increase in neonatal mortality since post-neonatal mortality remained unaffected. From 2008 to 2016, IMR and NMR both increased by 57%.

A major finding of our analysis was the collapse of the five-decade accelerating improvement in infant death rates caused by the great austerity between 2008 and 2016. At the end of 2008, Greece was hit by an unprecedented economic crisis that had a marked impact on population health, and perinatal rates, as the most sensitive to the impact of social determinants of health, were the first to be affected [13]. Thus, an increase in stillbirths was associated with the economic downturn [14,15], but early reports on infant mortality were inconclusive [13,16-18], prompting a comprehensive nationwide time-trend analysis. The present study, with the longest follow-up period of national data as contrasted with previous studies, highlighted comprehensively the dramatic impact of the great recession on IMR, showing the relative increase, a statistically significant trend since 2008, and the unprecedented upward trend after five decades of increasing improvement. In a study of trends in IMR in Greece for the years 2000-2016, a reverse in decreasing trends of IMR and NMR was observed from 2009 to 2016, without significant change for the PNMR, with higher than expected values based on the 2000-2008 trends of 26% and 34% for the IMR and the NMR, respectively [19]. Significant associations of IMR with sociodemographic factors, including rural residence and human development index, were reported in another study that explored the IMR and NMR trends in Greece during 2004-2016 [10].

**Figure 10:**
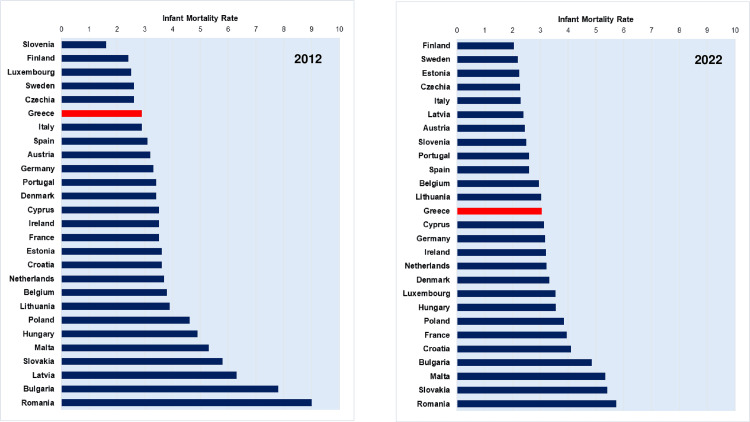
Infant mortality rates (per 1,000 live births) in the European Union countries, 2012 and 2022. Data were derived from Eurostat [20].

Furthermore, our analysis showed that the rising trend of the IMR due to economic austerity was interrupted in 2016, and since then the rate has shown a significant declining trend, which, however, has made the IMR less favorable than in the pre-crisis period. Moreover, the downward trend of the IMR has been relatively inadequate since Greece's position has deteriorated in the international ranking, falling from 6th place in 2012 to 13th place in 2022 among the countries of the European Union [20] (Figure 10).

Noteworthy, IMR was also negatively affected in other southern European countries. However, in Spain, Portugal, and Italy, the declining rate was attenuated, while Greece only saw a rising trend following the 2008 financial crisis. Additionally, there was a significant correlation between IMR and socioeconomic factors in all four countries [9].

Infant mortality has shown a remarkable decline worldwide in the past few decades. In Italy, there was a 54% decline in IMR from 1991 to 2009 [8]. The global decline from 1990 to 2010 was 57% for NMR and 62% for PNMR [1], and estimates for 2013 suggest that global NMR was 18.4 per 1,000 live births, down from 48.2 per 1,000 live births in 1970, and PNMR was 13.2 per 1,000 live births in 2013, as compared with 48.1 per 1,000 live births in 1970 [2]. Neonatal deaths fell by approximately 42% from 1990 to 2.6 million in 2015 [3], while NMR dropped 52% between 1990 and 2019, from 36.6 to 17.5 per 1,000 live births [21]. The considerable success in enhancing infant survival rates was a result of implementing public health and sanitation measures, expanding healthcare access and vaccination coverage, improving infection control for diseases such as malaria and HIV, and providing nutritional support [1-3,21-23]. 

It was estimated that in 2019, the underlying causes for nearly 70% of global neonatal deaths were preterm births (36%), intrapartum-related complications (24%), and congenital anomalies (10%) [24]. The group most at risk for death in the first year of life are small, vulnerable newborns, i.e., those who are preterm, have a low birth weight, or are small for gestational age [25-27]. The high incidence of preterm birth is the leading cause of infant death in the United States and the main reason behind the country's unfavorable ranking in infant mortality among developed countries [5,7,28,29]. Premature births have been reported as the leading cause of infant mortality in Greek-based studies [30,31]. Preterm birth rates are extremely high in Greece [32-34], and represent a major challenge for achieving further reductions in infant mortality in the country.

There are many more prospects for immediate reductions, sub-doubling or even sub-tripling, of infant mortality rates in Greece. A global analysis suggests that an optimum of 0.80 per 1,000 live births is a feasible target for NMR if preventable causes are successfully addressed [11]. The challenge of prenatal medical care is the prevention and management of the major obstetrical syndromes, including preterm labor, fetal growth restriction, and pre-eclampsia, which constitute the core of the etiology of vulnerable newborn births [35], as well as the timely diagnosis of congenital anomalies. Furthermore, the reverse trajectory of infant mortality due to the deterioration of socio-economic conditions in the period of economic crisis clearly shows that the implementation of effective public health policies targeting unimpeded access to public perinatal health services, as well as social support and improvement of the socio-economic conditions of the most deprived social groups, are additionally required.

The strengths of the current study lie in the utilization of official national data sourced from birth and death certificates that are legislatively mandated to be completed accurately, alongside an extensive follow-up period that surpasses earlier Greek-based published studies, thereby enabling comprehensive observation of temporal patterns. The main limitation of the present study is the absence of a linkage between the birth and death certificate data used to calculate the mortality rates in Greece. Furthermore, this study was based on nationwide registry data. Therefore, it was not possible to reveal geographical variations as well as the effect of sociodemographic factors, whereas future analysis of trends in the biological causes of infant deaths is also essential.

## Conclusions

The results of the present time-trend analysis of nationwide data highlighted the substantial progress achieved for the infant and neonatal death rates in Greece, and even more so for post-neonatal mortality, reflecting the country's upward trend in health and socioeconomic status. Gains in infant survival were larger in each sub-period until 2008, when the country was plunged into a labyrinth of austerity and social cuts. The recovery of IMR after 2016 is encouraging, but the rate still falls short of the pre-crisis period. There is great scope for further reducing preventable infant deaths by ensuring equity in healthcare services and addressing the social determinants of perinatal health.
